# Effect of the transpulmonary pressure on the lungs’ vibroacoustic response: a first numerical perspective

**DOI:** 10.3389/fdgth.2025.1434578

**Published:** 2025-04-04

**Authors:** Arife Uzundurukan, Sébastien Poncet, Daria Camilla Boffito, Philippe Micheau

**Affiliations:** ^1^ Centre de Recherche Acoustique-Signal-Humain, Université de Sherbrooke, Sherbrooke, QC, Canada; ^2^ Department of Chemical Engineering, École Polytechnique de Montréal, Montréal, QC, Canada

**Keywords:** digital twin of the lungs, Biot's theory, respiratory therapy, HFCC, vibro-acoustic therapy, transpulmonary pressure (PL), human thorax model

## Abstract

In the high-stakes environment of intensive care units (ICUs), managing transpulmonary pressure is crucial for providing breathing assistance to intubated patients, particularly when combining this intervention with respiratory therapy, such as high-frequency chest compression (HFCC). Despite the complexity of lung tissues, a computed tomography-based finite element model (CT-FEM), guided by Biot's theory, can be employed to numerically predict their vibroacoustic behavior at low frequencies, where the properties of the lungs align with the theory's principles. In this work, one aims to develop an analytical model of the lungs for two different levels of transpulmonary pressure—10 cm H_2_O (inflated lungs) and 20 cm H_2_O (healthy lungs)—to examine the poroviscoelastic behavior of the lungs and evaluate the generated analytical model using a CT-FEM of the human thorax like a digital twin of the human thorax. Biot's theory was utilized to predict the complex-valued shear wave speed, as well as the fast and slow compression wave speeds, across a frequency range between 5 and 100 Hz. The analytically computed values were tested using a previously validated CT-FEM of the human thorax to compare respiratory therapy outcomes for intubated patients under different transpulmonary pressure levels. Besides the frequency response function of the thorax, the kinetic energy density and the strain energy density were compared for these pressure levels. The CT-FEM demonstrated that all peak points fall within the range of 20–45 Hz; therefore, this range might be considered in ICUs settings. Kinetic energy density was nearly 2.2 times higher, and strain energy density was 1.46–1.26 times higher at the first and last peaks, respectively; therefore, inflated lungs experienced greater effects than healthy ones under the same respiratory therapy conditions. Overall, this study highlights how different transpulmonary pressures affect HFCC therapy, offering insights into gentle and effective conditions for intubated patients in ICUs while revealing the lungs’ 3D responses by integrating analytically predicted shear wave speed, fast and slow compression wave speeds.

## 1 Introduction

The spectrum of potential risks for patients in intensive care units (ICUs) requiring intubation, spans from gagging, choking, bleeding, and soft tissue damage to sore throat, hoarseness, and oral trauma (1). For instance, in a prospective observational study conducted from October 2018 to July 2019, nearly 3k patients across 197 sites in 29 countries, at least one major clinical event occurred after intubation in 45.2% of patients, including cardiovascular instability in 42.6%, severe hypoxemia in 9.3%, and cardiac arrest in 3.1% (2). A conscientious appraisal of these risks, coupled with the implementation of requisite measures, is paramount for ensuring patient safety and well-being (3, 4). Furthermore, monitoring transpulmonary pressure helps ICUs clinicians optimize ventilator settings and assess lung function in critically ill individuals (3, 5). Therefore, many critical settings, such as transpulmonary pressures, are the hottest research topics in ICUs, which aim not only to enhance our understanding of the pathophysiology of acute respiratory failure but also to improve clinical outcomes (5). Intubated patients, who have a tube assisting their breathing, may necessitate cautious consideration for implementing vibroacoustic chest physiotherapy (CPT) in critical care settings like ICUs (6). CPT is a technique utilizing low-frequency vibrations to induce stimulation of the rheological properties of the bronchial mucus and supply easier relaxation in the airways (7). Vibroacoustic therapy, an indispensable component of respiratory care for critically ill patients in the ICU, has emerged as a promising combination with ventilation in terms of being both safe modality (8) and supportive for the effective full range of the secretion clearance (9). This innovative approach involves the use of mechanical vibrations to stimulate various physiological responses in the body, promoting potential therapeutic benefits by making the mobilization of secretions easier, which are then cleared by cough or by suction in the case of intubated patients (10, 11). Therefore, it is obvious that the combination of intubation and vibroacoustic therapy applied to the dorsal part of the thorax is well-suited for patients with muco-obstruction in the airways by minimizing the discomfort associated with the risky conditions while increasing the efficiecy of the mucus drainage.

An effective method for administering therapy involves the utilization of vibroacoustic airway clearance devices (ACDs), such as the high-frequency chest compression (HFCC) devices (7, 12). These devices deliver gentle oscillations to the chest surface, providing a tailored and regulated treatment approach (7, 11). This approach proves beneficial for facilitating sputum expectoration, contributing to the stabilization or enhancement of respiratory function, and augmenting airflow in regions characterized by low lung volumes (10, 11).

The lungs are made of soft biological tissue, vasculature, as well as millions of microscopic air sacs connected through a complex branching airway structure (13). Therefore, the lungs are highly heterogeneous, combining gas linked through a complex and tortuous network of channels and microscopic sacs (14). Furthermore, they include flowing non-Newtonian liquid, blood, a complex network of vessels, and a viscoelastic soft tissue structure exhibiting nonlinear behavior under large deformation (15). It is apparent that pulmonary conditions can result in considerable changes in the stiffness or density of the lungs, either locally or diffusely (16). In the case of fibrotic lung, for instance, the stiffness of lung parenchyma rises in proportion to the severity of fibrosis (17). Conversely, asthma exhibits an elevated bronchoconstriction level, which correlates with an increase in parenchymal shear modulus (18). In cases of emphysema, the lung experiences a reduction in stiffness due to the restructuring of collagen fibers (19). Furthermore, it is an established fact that gradually increasing inhalation pressure significantly improves lungs’ elasticity (20) and the effect of tissue and model uncertainties on the lung response (21). However, transpulmonary pressure influences the physical features of both the elastance and air ratio (22). Therefore, in order to supply comprehensive lung feature variations, transpulmonary pressure is taken as a comparison variable parameter in this study.

Although detecting these changes is often challenging with conventional imaging, the transpulmonary pressure is central to comprehending respiratory mechanics, which represents the pressure differential across the lung and drives pulmonary ventilation for different conditions (23). It could be used to determine the physical properties of the lungs for numerical studies. A transpulmonary pressure ranging from approximately 25–30 cm H_2_O is associated with full inflation of the normal lung, leading to the recommendation of not being more than 30 cm H_2_O as an estimate of the elastic distending pressure (24). Suggested upper limits for tidal changes in transpulmonary pressure are 15–20 cm H_2_O for patients with homogeneous lung parenchyma (normal, post-surgical patients) and 10–12 cm H_2_O for in patients with inhomogeneous lung parenchyma (acute respiratory distress syndrome) in clinical settings (5). Therefore, in this study, transpulmonary pressure levels of 10 and 20 cm H_2_O were selected to see the difference between the patients and healthy lungs with vibroacoustic therapy for numerical studies. This kind of numerical study used for CT-FEM was seen as a new simulation technology that can revolutionize respiratory medicine while decreasing the intubation risks, especially for prolonged ventilation.

The study of the mechanical properties of the lungs during breathing is of utmost importance in determining physiotherapy conditions as well as clinical diagnostics (25). As the primary organs involved in respiratory mechanics, the lungs’ behaviour of shear and compression waves, known as also dilatational and rotational waves, in lung tissue provides valuable insight for medical professionals in managing conditions such as asthma, COPD, and respiratory distress syndromes (26). Incorporating precise data into biomechanical models allows for a more accurate representation of tissue mechanics, which in turn enables the development of advanced medical devices and treatments (11). A comprehensive understanding of the behavior of shear and compression waves in biomedical materials is of paramount importance in advancing medical diagnostics, devising effective treatment strategies, and conducting research on respiratory health and diseases.

The inquiry into poroelastic wave propagation within poroviscoelastic, fluid-saturated lung material intricately integrates effective medium theory to model macroscopic behavior and Biot's theory to describe the interaction between fast and slow compressional waves, shear waves, and the dynamic coupling of solid and fluid phases in lung tissues (27). The effective medium theory has been used to model lungs in acoustical studies since the 1980s (27, 28). This theory could be applicable in the frequency range of 100–2,000 Hz unsuitable for frequencies below 100 Hz (27, 29). This stems from the premise that the swift oscillations prompted by the compression wave impede the airflow within the lungs from transitioning between different regions (30). Moreover, a homogeneous isotropic material model resembles water uniformly interspersed with small gas bubbles, facilitating the calculation of compression wave behavior across the audible frequency spectrum (31, 32). However, the Biot's theory is more accurate and robust than the effective medium theory for reliable results in HFCC studies as it is applicaple for the frequency ranges below 100 Hz. Furthermore, the Biot's theory has been used to describe the wave behavior in the heterogeneous dynamic porous saturated medium by neglecting the microscopic level and averages of the corresponding microscopic quantities of the constituents Poiseuille flow (33) and breakdown of Poiseuille flow (34).

In order to study HFCC numerically, in addition to a realistic geometry of the internal organs, knowing their material properties is crucial for accurate results in FEM. Experimental *in vivo* studies cannot replicate human respiratory organs. To overcome this limitation, numerical models can provide unbiased solutions that are independent of external effects. By using 3D FEM models, one can improve the understanding of relevant vibroacoustic wave transmission phenomena (30). Therefore, the modelling of the complex material properties of the human respiratory organs is one of the most critical parts of FEM. However, in the literature, scientists focus on the high-frequency range to detect the parenchyma in pulmonary diseases (27), and no study has been conducted at low frequencies for the lungs’ material features and behavior. Further, there are contrasting data and opinions about the working conditions of these devices. However, in our previous study, we found two apparent resonance frequencies at 28 and 41 Hz for the human thorax in the 5–100 Hz range (11), which are confirmed by two experimental studies (35, 36).

Experimental data indicate that an increase in transpulmonary pressures supplies benefits from 10 to 20 cm H_2_O (37). However, another study consisting of 66 patients indicates that receiving mechanical ventilation and HFCC together with the aim of increasing pressure doesn't have any therapeutic benefit (38). Vibration therapy is pervasively recognized as effective for pulmonary secretion clearance (11), albeit with a critical caveat—limited understanding regarding the different frequency ranges with respect to the impact on the lungs at different transpulmonary pressure levels. Furthermore, in terms of the lung analytical modelling, only Siklosi et al. (39) focused on 500–5,000 Hz, and Peng et al. (40) on 100–800 Hz, providing purely analytical models. Then there is no study in the low frequency range (below 100 Hz), which is is used for HFCC (11). Therefore, CT-FEM has the potential to transform respiratory medicine by addressing the significant gap in the literature regarding analytical models and their validation, and enhancing reliability by accounting for varying lung conditions based on transpulmonary pressures and low-frequency ranges. Addressing this gap represents a clinical need, as such models provide insights into lung mechanics under varying conditions, such as transpulmonary pressure and external excitations. Digital twins of the lungs, analytically developed alongside 3D CT-FEM advancements, can enhance diagnostics and enable personalized treatments for respiratory disorders. This lacuna in knowledge constitites the motivation of this study, aspiring to shed light on the mechanical ramifications of chest wall vibration in healthy individuals, alongside an exploration of transpulmonary pressures and HFCC therapy settings together as a combined settings.

Here, we aimed to develop a tractable set of equations to render microscopic heterogeneous features of the lungs possible to represent in macroscopic material properties and test them. It is the first time the physical properties of the lungs were analytically modelled by Biot's theory, culminating in comparing predicted wave attributes, namely wave speeds and attenuation at different pressures at a low-frequency range. Another novelty of the present study lies in testing the analytically calculated lung material features as input to a previously validated CT-FEM of the human thorax. This approach allowed to generate a visualization comparison of the lungs at different frequencies and transpulmonary pressure levels as a combined therapy. To bring more clarity, both 1D with a homogenously distributed average of 5k points mean and 3D results for kinetic and elastic strain energy density were evaluated at their peak points. Here, we contributed to respiratory studies determining the material properties of the lungs for numerical studies and providing a visual illustration of their behavior, resulting in an heightened understanding of lung responses at peak frequencies.

## 2 Methodology

Two key circumstances prevalent in clinical settings were identified as exerting significant influence on the material properties of the lungs and resulting in distinct responses: lungs afflicted with inflation experiencing a transpulmonary pressure of 10 cm H_2_O, and normal lungs subjected to a transpulmonary pressure of 20 cm H_2_O. These two conditions were modeled with the Biot's theory described in Section 2.1. Afterwards, these analytically modelled lungs at different pressure conditions, and their vibroacoustic lung behavior, were tested and compared by using a previously validated CT-FEM described in Section 2.2.

### 2.1 Biot theory

The Biot's theory describes the propagation of waves in a solid porous structure fully saturated with fluid, wherein it disregards the microscopic level and posits the applicability of continuum mechanics to measurable macroscopic quantities (33, 34). Here, one follows the Biot's theory (34), in which the breakdown of the Poiseuille flow exists. Lung structure depends on various variables, but the most obvious of which is the air inside. This air fills the alveoli and puts pressure on them from the inside, affecting many material properties such as pore size, permeability, and shear stress (14). The material properties are extracted from an experimental study, in which the chest cavity was surgically opened and pleural pressure adjusted to atmospheric pressure and the transpulmonary pressures were maintained at 10 and 20 cm H_2_O (27).

The Biot's theory is applied to the lungs by assuming a homogeneous isotropic poroviscoelastic material with small deformations for HFCC therapy to represent ACDs effect. Neglecting the initial conditions, the Biot's equations as determined in Equations 1, 2 describe a frequency-dependent speed of sound at low frequency range inside the lungs, describing the dynamic behavior of the material in terms of macroscopic, space-averaged quantities such as acoustic pressure, elastic stress, and solid and fluid displacements. Here, Einstein summation notation is used to illustrate x, y, and z coordinates and subscript j denotes the partial derivative. They are written in the frequency domain to illustrate the results under harmonic oscillations (41):

(1) $$\begin{array}{c} \mu u_{i,jj}+\left(K_{b}+\frac{\mu }{3}\right)u_{\;j,ij}-(\alpha -\beta )P_{,i}+F_{e}=-\omega^{2}(\rho -\beta \rho_{f})u_{i} \end{array}$$

(2) $$\begin{array}{c} \beta p_{,ii}+\frac{\varphi^{2}}{R}\rho_{f}\omega^{2}p+\rho_{f}j\omega r_{g}=-\rho_{f}\omega^{2}(\alpha -\beta )u_{i,i} \end{array}$$

where *u* is the steady-state dynamic oscillatory displacement, *p* represents the dynamic pressure of the air in the lungs in the frequency domain. *α*, *β*, and *R* are the coupling parameters between the lung parenchyma and air (described in Equations 3–5, respectively). Here, ρ is the lung density and $\rho_{f}$ is the air density inside the lungs. *F_e_* is the external input of force, *ω* is the angular velocity, where *jω* denotes a time derivative, $r_{g}$ indicates the rate of introduction of gas per unit volume, *K_b_* is the drained bulk modulus, and μ is the complex shear viscoselasticity, which defines the complex shear wave speed.

(3) $$\begin{array}{c} \alpha =1-\frac{K_{b}}{K_{sk}} \end{array}$$

(4) $$\begin{array}{c} \beta =\frac{\kappa \rho_{f}\varphi^{2}j\omega }{\varphi^{2}+j\omega \kappa (\rho +\varphi \rho_{f})} \end{array}$$

(5) $$\begin{array}{c} R=\frac{\varphi^{2}K_{f}K_{sk}^{2}}{K_{f}(K_{sk}-K)+\varphi K_{sk}(K_{sk}-K_{f})} \end{array}$$

where *K_sk_* is the skeleton bulk modulus, *K_f_* is the fluid bulk modulus, and κ is the permeability. One assumes that *K_f_* = *nP*, where the polytropic constant *n* is fixed to 1 as an isothermal process (42) in the low frequency range.

(6) $$\begin{array}{c} \rho =(\tau -1)\rho_{f}\varphi \end{array}$$

Here τ denotes the tortuosity at low frequencies (43) and plays a crucial role to determine ρ, as well as $\rho_{f}$ and volume fraction (φ) described in Equation 6. It is a geometric factor influenced by the pore structure, with the pore space in the lungs being approximated as a meandering cylindrical channel network with a variable diameter.

(7) $$\begin{array}{c} \kappa =\frac{\kappa_{p}}{\nu_{f}} \end{array}$$

where the permeability of the porous medium $\left(\kappa_{p}\right)$ is related to volume fraction (φ), specific area of the porous section (*A*), and tortuosity (τ) as illustrated in Equations 7, 8:

(8) $$\begin{array}{c} \kappa_{p}\propto \varphi^{3}\frac{A}{\tau } \end{array}$$

The complex-valued fluid viscosity $\nu_{f}\;$ is defined as in Equation 9:

(9) $$\begin{array}{c} \nu_{f}=F(\Theta )\eta_{f} \end{array}$$

where F(Θ) is the frequency correction function, which is necessary as the fluid viscosity $\left(\eta_{f}\right)$ is dynamic as described in Equation 10 (33). The Poiseuille flow is valid when the duct wall is at rest, but when the fluid and the solid skeleton oscillate at a given frequency, there is a deviation from the Poiseuille flow and the dynamic viscosity of the fluid is multiplied by a frequency correction parameter (Θ) and frequency correction factor *T*(*Θ*) as determined in Equations 11, 12, respectively:

(10) $$\begin{array}{c} F(\Theta )=\frac{1}{4}\left(\frac{\Theta T(\Theta )}{1+2j\frac{T(\Theta )}{\Theta }}\right) \end{array}$$

where

(11) $$\begin{array}{c} \Theta =\frac{d}{2}\sqrt{\frac{\xi \omega \rho_{f}}{\eta_{f}}} \end{array}$$

Here, ξ is the sinuosity function determined as $\xi =\sqrt{\tau }$, and *d* is the diameter of the alveolar.

(12) $$\begin{array}{c} T(\Theta )=\frac{-\sqrt{-j}J_{1}\left(\sqrt{-j}\Theta \right)}{J_{0}\left(\sqrt{-j}\Theta \right)} \end{array}$$

where *J*_1_ and *J*_0_ are the Bessel functions of the ﬁrst kind of orders 1 and 0, respectively. So, at high frequencies, one gets:

(13) $$\begin{array}{c} F(\Theta )\to \Theta \frac{\frac{1+j}{\sqrt{2}}}{4} \end{array}$$

A phase difference exists between the velocity and the friction force. At constant velocity, the friction force grows with the square root of the frequency and maintains a 45-degree phase offset from the velocity (34). When Equations 1, 2 are coupled to the compression behavior, it gives:

(14) $$\begin{array}{c} \left(K_{b}+\frac{4\mu }{3}\right)u_{i,jji}-(\alpha -\beta )p_{,ii}=-\omega^{2}(\rho -\beta \rho_{f})u_{i,i} \end{array}$$

where

(15) $$\begin{array}{c} u_{i,jj}=-\omega^{2}(\rho -\beta \rho_{f})u_{i} \end{array}$$

Here, one assumes that dilation is zero $\left(u_{i,i}=0\right)$. Then, *c_s_* and *k_s_* represent the shear wave speed and shear wave number, respectively, and write as follows. Then, *c_s_* and *k_s_* represent the shear wave speed and shear wave number, respectively, and write as follows in Equations 16, 17, respectively:

(16) $$\begin{array}{c} c_{s}=\sqrt{\frac{\mu }{\rho -\beta \rho_{f}}} \end{array}$$

(17) $$\begin{array}{c} k_{s}=\frac{\omega }{c_{s}} \end{array}$$

Here, the real part of *k_s_* governs the shear wave speed and the imaginary part defines the attenuation.

As for the calculation of the compression waves, Equations 14, 15 could be written as in Equations 20–25, respectively:

(18) $$\begin{array}{c} \bar{\alpha }{u^{\prime \prime }}_{x}-\bar{\beta }{\;p}^{\prime }={\left(\;j\omega \right)}^{2}\bar{\Gamma }u_{x} \end{array}$$

(19) $$\begin{array}{c} \bar{\Delta }{\;p}^{\prime \prime }-{\left(\;j\omega \right)}^{2}\bar{\varepsilon }p={\left(\;j\omega \right)}^{2}\bar{\Omega }{u^{\prime }}_{x} \end{array}$$

where the determined constants, $\bar{\alpha }$,$\;\bar{\beta }$, $\bar{\Gamma }$, $\bar{\Delta \;}$, $\bar{\varepsilon }$, and $\bar{\Omega }$, are regulated as follows:

(20) $$\begin{array}{c} \bar{\alpha }=K_{b}+\frac{4\mu }{3} \end{array}$$

(21) $$\begin{array}{c} \bar{\beta }=\alpha -\beta \end{array}$$

(22) $$\begin{array}{c} \bar{\Gamma }=\rho -\beta \rho_{f} \end{array}$$

(23) $$\begin{array}{c} \bar{\Delta }=\beta \end{array}$$

(24) $$\begin{array}{c} \bar{\varepsilon }=\frac{\varphi^{2}}{R}\rho_{f} \end{array}$$

(25) $$\begin{array}{c} \bar{\Omega }=\rho_{f}(\alpha -\beta ) \end{array}$$

Further, ′ and ″ denote the first and second order derivatives, respectively, according to the *x* direction. Therefore, with respect to the oscillation with increase in oscillation (ω) could be considered for the displacement and pressure calculation as follows:

(26) $$\begin{array}{c} u_{x}=u_{0}e^{\;j(\omega t-kx)} \end{array}$$

(27) $$\begin{array}{c} \;p=p_{0}e^{\;j(\omega t-kx)} \end{array}$$

When Equations 26, 27 are replaced in Equations 18, 19, the matrix form of the equations is obtained as given in Equation 28:

(28) $$\begin{array}{c} \left[\begin{array}{cc} \bar{\alpha }k^{2}-\bar{\Gamma }\omega^{2} & -j\bar{\beta }k\\ \;j\bar{\Omega }\omega^{2}k & \bar{\Delta }k^{2}-\bar{\varepsilon }\omega^{2} \end{array}\right]\left[\begin{array}{c} u_{x}\\ p \end{array}\right]=\left[\begin{array}{c} 0\\ 0 \end{array}\right] \end{array}$$

The quadratic equation of the matrix form, therefore, is obtained as:

(29) $$\begin{array}{c} \bar{\alpha }\bar{\Delta }k^{4}-(\bar{\alpha }\bar{\varepsilon }+\bar{\Gamma }\bar{\Delta }+\bar{\beta }\bar{\Omega })\omega^{2}k^{2}+\bar{\Gamma }\bar{\varepsilon }\omega^{4}=0 \end{array}$$

Here, Equation 29 results in 2 different values, *k_ps_* and *k_pf_*, which are the slow and fast compression wave numbers, respectively the used material features of the lungs used in the calculation are given in Table 1.

**Table 1:** Material properties used in CT-FEM for the internal organs.

List	Properties	Value	References
Lung properties	Air density, *ρ*_f_	1.2 kg.m^−3^	(27)
	Solid density, *ρ*_t_	1,000 kg.m^−3^	(52)
	Tortuosity, *τ*	1.33	(53)
	Polytrophic constant	1	(27)
	Dynamic air viscosity, *η*_f_	1.82 × 10^−5^ Pa.s	(27)
	Solid bulk modulus, *K*_t_	2.2 × 10^9^ Pa	(52)
Lung properties at 20 cm H_2_O	Air volume fraction, φ	0.71	(27)
	Air pressure, *P*	1.03 × 10^5^ Pa	(27)
	Permeability of porous medium, *κ*_p_	25 × 10^−12^ m^2^	(27)
	Pore radius, *r*	0.225 mm	(54)
	Solid skeleton bulk modulus, *K*_sk_	8.26 × 10^3^ Pa	(27)
	Solid shear modulus, *μ*_s_	1,400 + 5.78 (*jω*)^0.5^	(27)
	The total volume of the lung, *V*_L_	1,141 cm^3^	(27)
Lung properties at 10 cm H_2_O	Air volume fraction, φ	0.57	(27)
	Air pressure, *P*	1.02 × 10^5^ Pa	(27)
	Permeability of porous medium, *κ*_p_	13 × 10^−12^ m^2^	(27)
	Pore radius, *r*	0.2 mm	(54)
	Solid skeleton bulk modulus, *K*_sk_	4.68 × 10^3^ Pa	(27)
	Solid shear modulus, *μ*_s_	790 + 1.23 (*jω*)^0.5^	(27)
	The total volume of the lung, *V*_L_	770 cm^3^	(27)
Airways	Young's modulus—Real part, *E*_1_	0.28 MPa	(29)
	Young's modulus—Complex part, *E*_2_	0.124 MPa	(29)
	Density, *ρ*_f_	1,000 kg.m^−3^	(29)
	Poisson's ratio, *ν*	0.49998	(29)
Soft tissue	Lamé parameter—Real part, *λ*_1_	2.6 GPa	(52)
	Lamé parameter—Complex part, λ_2_	0	(52)
	Shear modulus—Real part, *μ*_1_	2.5 kPa	(52)
	Shear modulus—Complex part, *μ*_2_	5 Pa.s	(52)
	Density, *ρ*_st_	1,000 kg.m^−3^	(52)
Osseous	Lamé parameter—Real part, *λ*_1_	2.6 GPa	(52)
	Lamé parameter—Complex part, *λ*_2_	0	(52)
	Shear modulus—Real part, *μ*_1_	10 × 10^6^ MPa	(52)
	Shear modulus—Complex part, *μ*_2_	20 Pa.s	(52)
	Density, *ρ*_o_	1,500 kg.m^−3^	(52)

The slow compression wave speed (*c_ps_*) and fast compression wave speed (*c_pf_*) are calculated as follows in Equations 30, 31, respectively:

(30) $$\begin{array}{c} c_{\;ps}=\frac{\omega }{k_{\;ps}} \end{array}$$

(31) $$\begin{array}{c} c_{\;pf}=\frac{\omega }{k_{\;pf}} \end{array}$$

where the imaginary part of fast compression wave number (*k_pf_*) and slow compression wave number (*k_ps_*) defines the attenuation as function of frequency.

As the last step, the analytically modelled the $c_{\;ps}$ value was integrated, along with $c_{s}$ and ρ, the density of the lungs, to CT-FEM as key material properties for both 10 and 20 cm H_2_O transpulmonary pressures. These additions enhance the model's accuracy by offering a more comprehensive representation of the lung's mechanical properties, thereby refining the simulation of wave propagation and tissue response under various conditions.

### 2.2 Testing of the analytically calculated transpulmonary pressures

**Figure 1:**
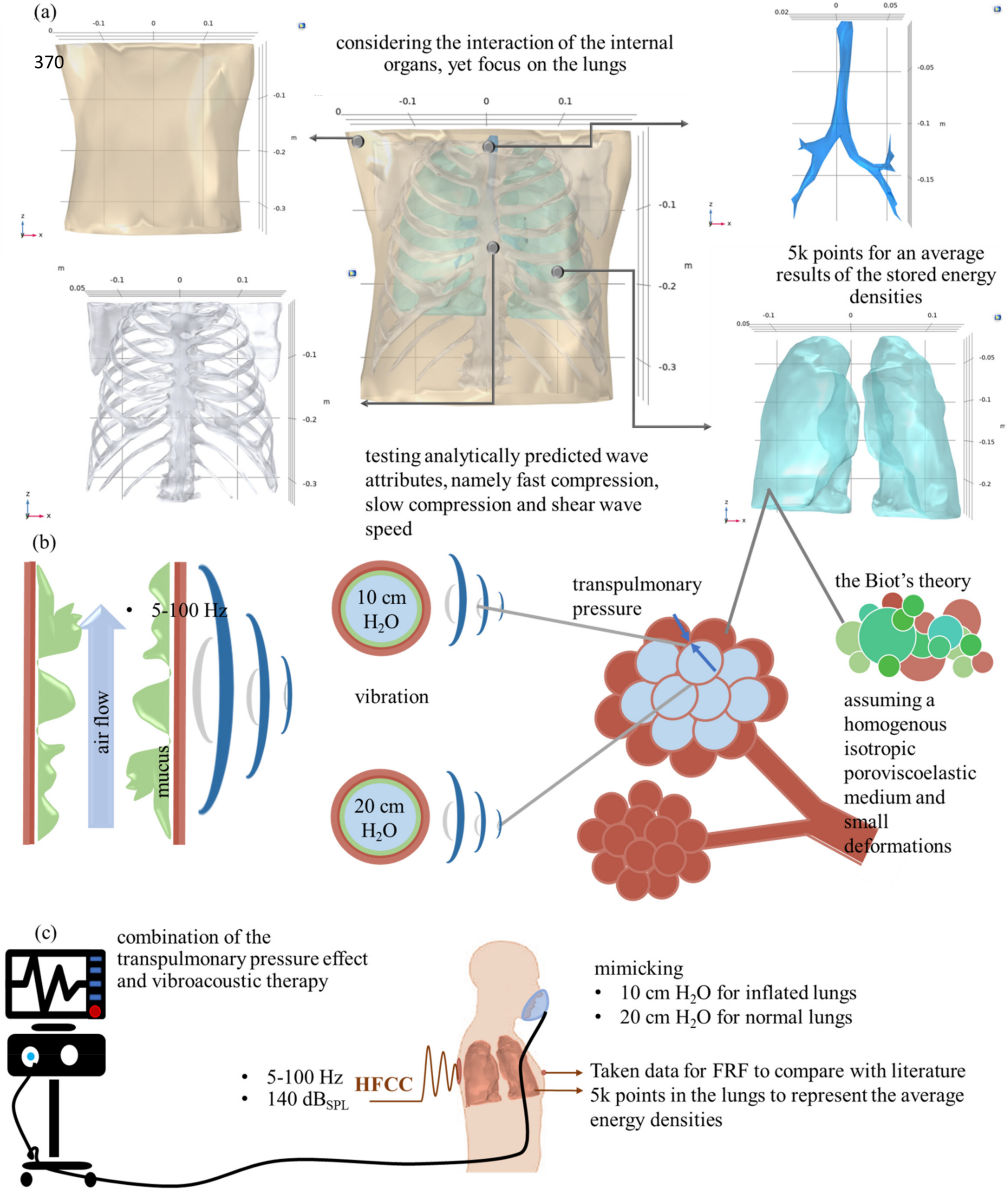
**(a)** 3D medical imaging of the assembled (46) and validated (11) human thorax; **(b)** transpulmonary pressure and vibrations on the alveola, and **(c)** representation of the combined treatment and boundary conditions, replicating inflated and normal lung conditions at 10 and 20 cm H₂O transpulmonary pressure levels and obtaining data for FRF and lung energy densities.

To accurately assess the external effect on the body, dorsal vibroacoustic therapy requires a realistic representation of human soft tissues, the rib cage, lungs, trachea, and bronchial structures. Although there is still a lack of full developed digital human twin, the CT-FEMs are currently used to model respiratory disease (11), road traffic accidents and ballistic impact (44), and epicardial pulsed electric field ablation (45). However, to guarantee the precision and dependability of the analytically developed lung material properties, it is imperative to test the calculated values in a formerly developed model for respiratory diseases (46), and validated for HFCC (11). Therefore, one developed a 3D medical imaging model of the assembled human thorax (46) and validated it (11), focusing on transpulmonary pressure and vibrations affecting the alveoli, and a combined treatment approach, as illustrated in Figure 1a. Therefore, this CT-FEM-based digital twin simulation incorporates predicted fast compression, slow compression, and shear waves, illustrating their interaction under varying transpulmonary pressures and providing valuable insights into lung mechanics under oscillatory forces.

In order to implement the therapy, boundary conditions are applied to the generated model, as illustrated in Figures 1a–c. In Figure 1a, an image of the soft tissue, ribcage, lungs, trachea, and overall assembled geometry is illustrated. For the mean stored energy densities of the lungs, average values are calculated from 4,948 points, randomly and evenly distributed for each frequency within the range of 5–100 Hz. All material properties are kept constant, except for the lungs, which vary depending on the transpulmonary pressure (10 or 20 cm H_2_O), as illustrated in Figure 1b in the alveoli and bronchiolar levels. This approach allows for a comparison of lung conditions at low frequencies. Figure 1c illustrates the boundary conditions of intubated patients with HFCC. A level of 140-decibel sound pressure level (dB_SPL_) is applied to simulate the shaker's working condition (radius of 0.04 m and applied force of 1 N) in the numerical model. The remaining boundary conditions are left unbound, following the approaches described in both experimental (35, 36) and numerical (11) studies. The frequency response function (FRF) of the human thorax was collected from front chest surface.

## 3 Results

### 3.1 Analytical modeling of the physical properties of the lungs

**Figure 2:**
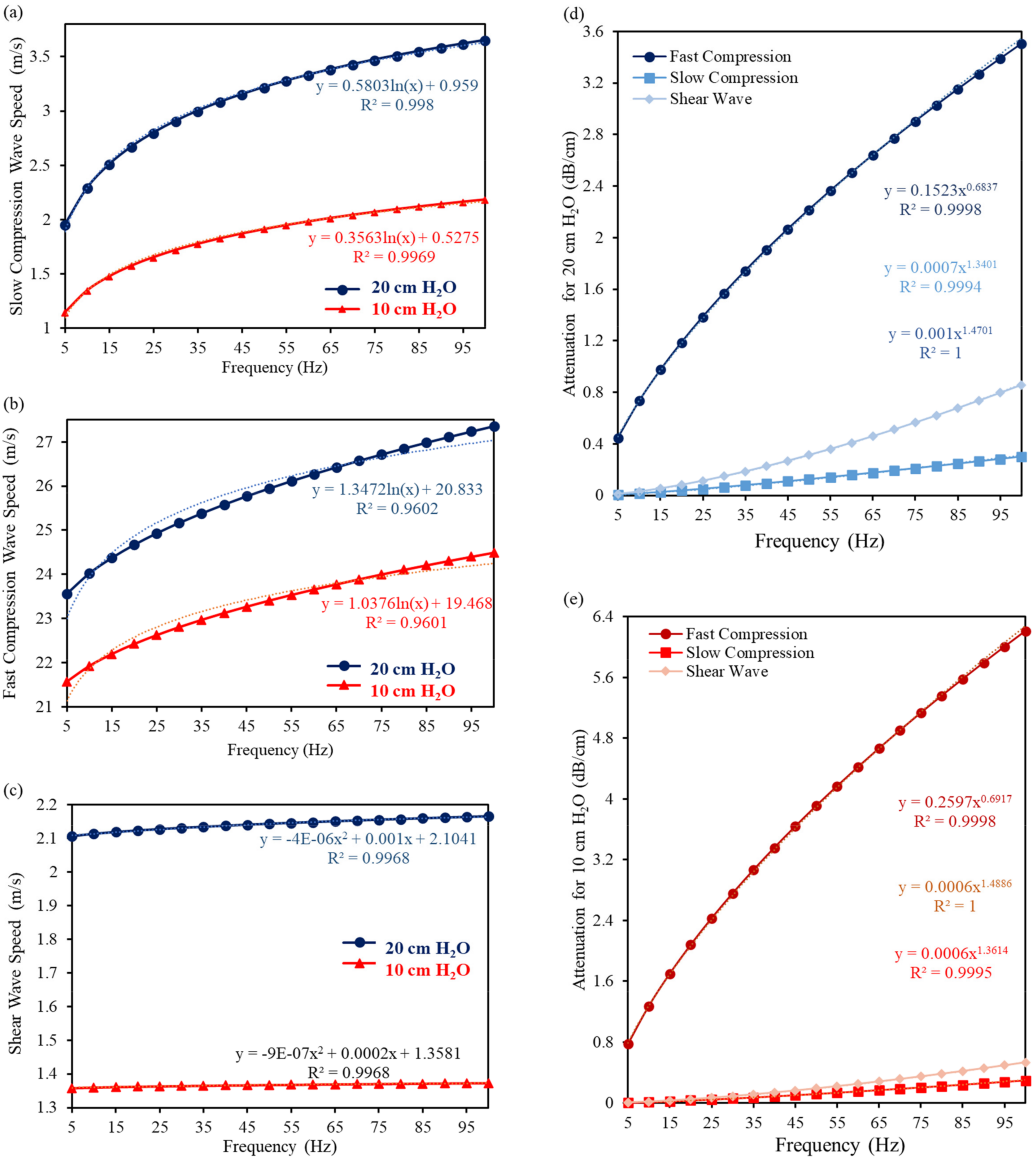
The analytical modelling of **(a)** fast compression wave, **(b)** slow compression wave, and **(c)** shear wave speed, as well as attenuations at **(d)** 20 cm H_2_O and **(e)** 10 cm H_2_O with respect to different frequencies.

The key aspects of the Biot's theory are applied to the lungs at both 10 and 20 cm H_2_O transpulmonary pressures, culminating in a comparison of predicted wave attributes by the analytical model, namely fast compression, slow compression and shear wave speed. Furthermore, attenuation is determined to see the effect of the HFCC working frequency on the lungs. The analytical modelling of fast, slow compression waves, and shear wave speed, as well as the attenuations at 10 and 20 cm H_2_O with respect to different frequencies are illustrated in Figures 2a–e, respectively.

As a result of the analytical modelling of the lungs material properties, it can be obviously seen from the graphs that both slow and compression waves are significantly frequency dependent, but slow compression is more dependent. According to the analytical calculations, *c_ps_* has an increasing rate of 46% and 48% for 20 and 10 cm H_2_O, respectively. From 5 to 100 Hz, the calculated *c_ps_* increases from 1.956 + 0.710*j* m/s to 3.652 + 0.911*j* m/s for 20 cm H_2_O, and 1.147 + 0.425*j* m/s to 2.186 + 0.582*j* m/s for 10 cm H_2_O.

As for *c_pf_*, the values increase by 13.9% and 11.9%, which raise from 13.9 23.560 + 1.075*j* m/s to 27.357 + 4.222*j* m/s for 20 cm H_2_O, and 21.568 + 0.830*j* m/s to 24.493 + 3.293*j* m/s for 10 cm H_2_O, from 5 to 100 Hz.

Although both slow and fast compression waves are influenced by an increasingly high frequency, the effect of this increasing frequency on the shear waves is relatively small, which is 2.7% and 1%, respectively. It increases from 2.106 + 0.017*j* m/s to 2.165 + 0.074*j* m/s for 20 cm H_2_O, and 1.359 + 0.004*j* m/s to 1.373 + 0.018*j* m/s for 10 cm H_2_O. Both fast compression and slow compression wave speeds raise significantly, as the tissue is becoming more responsive and transmits mechanical forces more efficiently at higher frequencies. These analytically calculated values are very close to those obtained in both numerical and experimental studies at 100 Hz (27). Unlike compression waves, shear waves typically exhibit a slightly different response to changes in applied frequency, while their velocity also increases but the rate of increase gets lower compared to compression waves. The exact key parameters used and predicted by the theories are given as Supplementary Material.

As for the attenuation, the amplitude tends to increase since the wave energy dissipation rises with respect to an increase in frequency and a decrease in the mean porosity (from 20 to 10 cm H_2_O); therefore, one could say that attenuation increases from healthy lungs to inflated one. Shear wave attenuation is mainly due to the lung shear viscosity. By increasing the frequency, the attenuation at 10 cm H_2_O is larger than that at 20 cm H_2_O as permeability (see Table 1 for exact values) is lower than that at 20 cm H_2_O.

### 3.2 Comparison of lungs modeled analytically at different pressures under HFCC

**Figure 3:**
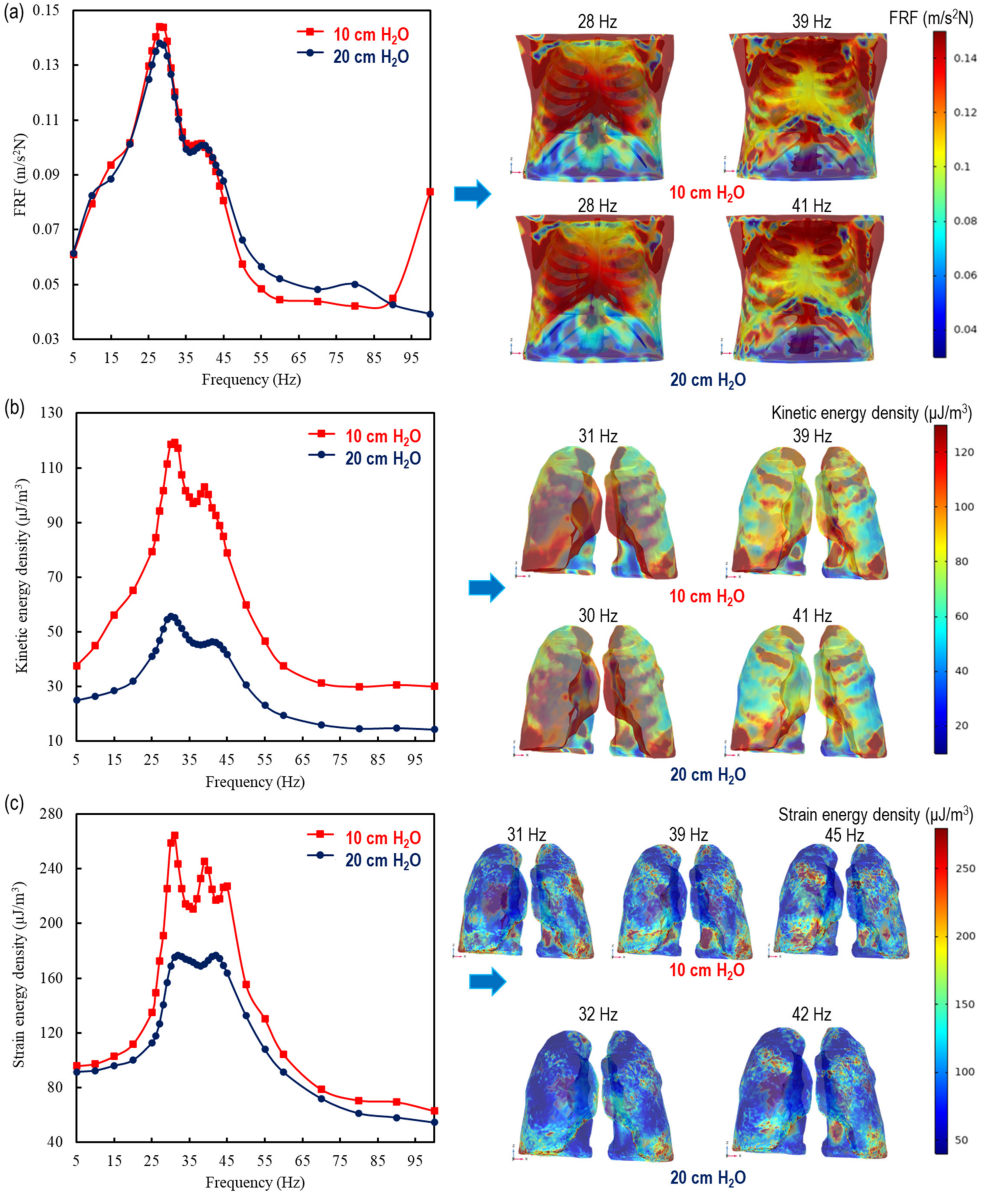
1D and 3D comparisons of the therapy effect by using analytically modelled lungs at 10 and 20 cm H_2_O for **(a)** the overall thorax in FRF (11) and the mean **(b)** kinetic energy density and **(c)** strain energy density in the lungs.

In this section we present a comparison between the therapy effects of analytically modelled lungs in 1D and 3D. The comparison is made for two different transpulmonary pressures 10 and 20 cm H_2_O. The analysis includes the overall thorax in FRF, as well as the mean stored kinetic energy density and strain energy density of the lungs in Figure 3. This comparison could provide valuable insights for improving therapy effectiveness and patient outcomes.

Thorax behaviour is similar at 10 cm H_2_O with the literature (11) 20 cm H_2_O transpulmonary pressure levels; 28 Hz, identitical frequency, at the major points and 39 and 41 Hz for the minor resonance frequencies, respectively. The major peaks in the human thorax, at 28 Hz, are found at 0.138 m/s^2^N (11) and 0.144 m/s^2^N for 20 cm H_2_O and 10 cm H_2_O, respectively. As for the minor peaks, they exhibit a similar behavior, at 40 Hz as 0.101 m/s^2^N (11) and 39 Hz as 0.102 m/s^2^N for 20 cm and 10 cm H_2_O, respectively. In addition to strengthening the lung analysis of these similarities, the results stem from being applied externally to the dorsal thorax, meaning that the investigated material includes not only the lungs but also the spine, rib cage, and surrounding tissues. Furthermore, beyond 100 Hz, there may be additional peak points, as indicated in the literature (47), and one can see this effect in the increase in the FRF value at 100 Hz for a transpulmonary pressure of 10 cm H₂O.

The lungs showcase a duality of peaks in mean kinetic energy density for both pressure conditions. For 20 cm H_2_O, the mean stored kinetic energy density in the lungs are found at 55.3 µJ/m^3^ and 46.3 µJ/m^3^ with a corresponding frequencys of 30 and 41 Hz, respectively, while at 10 cm H_2_O alongside distinct peaks are seen at 119 µJ/m^3^ and 103 µJ/m^3^ at a frequency of 31 and 39 Hz, respectively.

Despite the behavioural similarity in both the FRF of the thorax and stored kinetic energy density of the lungs, 2 different peaks for the thorax and 3 distinct peaks for the lungs were caught at both 10 and 20 cm H_2_O. At 10 cm H_2_O, the maximum strain energy densities are found as 264 µJ/m^3^, 245 µJ/m^3^, 227 µJ/m^3^ at 31 Hz, 39 Hz and 45 Hz, respectively while at 20 cm H_2_O, they get 180 µJ/m^3^ at both 32 Hz and 42 Hz.

## 4 Discussion

Vibration therapy has emerged as a salient asset due to its inherent safety, mitigating the proclivity for adverse effects commonly associated with more invasive therapeutic approaches. The intricate modeling of complex-valued lung shear and compression wave speeds within the 5–100 Hz frequency spectrum for HFCC constitutes a pivotal analytical cornerstone (11). Employing the Biot's theory for poroviscoelastic material homogenization unveils the frequency-dependent nature of lung physical properties at different transpulmonary pressures.

As the lung's structural changes highly depend on delivered inspired air to all lung units (14), the study scrutinized two distinct transpulmonary pressures at 10 and 20 cm H_2_O, revealing pronounced frequency dependence in fast and slow compression waves and shear waves. A significant increase in both fast and slow compression wave speeds, along with a slight increase in shear wave speed, was observed with increasing vibration frequency, as shown in Figure 2. Shear wave attenuation was primarily influenced by lung shear viscosity, while wave attenuation predicted by Biot's theory revealed notable differences. The reduction in compression wave intensity during propagation was driven by thermal dissipation under nonadiabatic conditions and viscous effects in both solids and fluids.

Table 1 indicates that the permeability of a porous material is 25 × 10^−12^ m^2^ and 13 × 10^−12^ m^2^ at 20 cm H_2_O and 10 cm H_2_O (27), respectively. Permeability, a measure of fluid passage through a porous material, plays a crucial role in attenuation, consistent with findings from the literature (48). The increased attenuation at higher frequencies can be attributed to enhanced friction between the alveolar duct walls and air, caused by a marked drop in permeability. Consequently, attenuation is higher at 20 cm H_2_O compared to 10 cm H_2_O, as shown in Figure 2, due to increased friction from reduced permeability. This decrease in permeability heightens the pressure gradient within the pore space, driving more air into the lungs. It is important to highlight that the relationship between lung permeability and attenuation is complex and influenced by various individual factors (49).

Furthermore, the analytically calculated waves were tested as inputs of the lungs’ material properties in previously modelled (46) and validated CT-FEM (11) of the human thorax. The vibroacoustic stimuli of the lungs in this model illustrated that even if the wave speed increases with respect to an increase in frequency, the maximum energy density remains between 20 and 45 Hz. The graphical representation of both energy densities depicted a significant downturn after approximately 40–45 Hz, which could be supported by the experimental outcomes in Ganesan et al.'s (1997) study (50), in which the authors found a cut-off frequency at 40 Hz due to lungs-ribcage interactions. This small variance between the numerical and experimental studies in the literature could be explained due to the interactions with the internal organs. Both soft tissues and rib cage envelope the lungs and have an effect on the transmission of the oscillation movements from the chest surface to the lungs. Furthermore, another study indicated that mucus viscosity tends to decrease by increasing in the frequency in this region and exhibits a shear thinning behavior (51). However, as there is no further effect of acoustic CPT on the lungs beyond 45 Hz, this reflects in a decrease of strain energy density (Figure 3). Thus, no enhanced mucus expectoration is expected at higher frequency. Therefore, one recommends that, in order to get the highest benefit from acoustic CPT, the frequency range for both transpulmonary pressures should be between 20 and 45 Hz for HFCC. In the literature, the efficacy of increased pressure support for intubated patients was demonstrated by (37). In the present case, augmenting pressure does not provide therapeutic benefitfmores (38). On the contrary, the increase in transpulmonary pressure leads to a decrease in both kinetic and elastic strain energy density in the lungs. This effect is more pronounced at 10 cm H_2_O compared to at 20 cm H_2_O.

In agreement with former findings from literature, the resonance frequencies are confirmed at 28 Hz and 41 Hz for the human thorax within the 5–100 Hz range (11). The capstone of this study is the innovative breakthrough involving the comparison of therapy at different transpulmonary pressures, which could be applicable in the high-stakes environment of ICUs. Moreover, the impact of respiratory therapy is significantly amplified in inflated lungs compared to healthy ones under identical operating conditions. Specifically, the kinetic energy density in inflated lungs is approximately 2.2 times greater, while the strain energy density exhibits a pronounced increase, ranging from 1.46 times higher at the initial peak to 1.26 times higher at the final peak. These findings underscore the heightened susceptibility and dynamic response of inflated lungs, offering deeper insights into the biomechanical effects of therapeutic interventions.

To sum up, this study did not only lay the groundwork for numerical investigations by providing the material characteristics of the lungs under various transpulmonary pressures and frequencies but also it revealed the influence of the transpulmonary pressure. This increase in transpulmonary pressure could indicate two different circumstances: (i) vibro-acoustic therapy for an intubated patient according to the difference in transpulmonary pressure and (ii) the difference in terms of vibro-acoustic therapy effect on both normal (20 cm H_2_O) and inflated lungs (10 cm H_2_O) (5). Furthermore, this technological advancement is set to significantly improve the clarity and understanding of the decrease in transpulmonary pressure. The transpulmonary pressure needed to maintain a particular lung volume is directly proportional to the lung elastance. In simple terms, if the lungs are stiffer (have higher elastance), more transpulmonary pressure is required to maintain the same volume. On the other hand, if the lungs are more compliant (have lower elastance), less transpulmonary pressure is needed to maintain the same volume. Therefore, it is obvious that the more compliant lungs stored more energy densities than the stiffer lungs. This holds significant implications and contributions in therapeutic paradigms on both vibro-acoustic and transpulmonary operating conditions for an intubated patient and the difference in the vibro-acoustic therapy effect on both normal and inflated lungs. The main limitation of the present study lies in disregarding the interactions with other internal organs (heart and diaphragm) and the respiratory cycle. The absence of mucus is another limitation of the present study as it can influence the pore viscoelasticity and the material properties of the lungs.

The spectrum of potential risks of respiratory disease spans from gagging, choking, bleeding, and soft tissues damage to sore throat, hoarseness, and oral trauma to increase the need of ICUs (1). HFCC has a great potential to decrease all these occurrences and this study appears really effective in the frequency range between 20 and 45 Hz for both inflated and normal lung conditions. On the other side, one should also consider that the highest energy density with a combined decrease in transpulmonary pressure may bring about other unpleasant effects. Furthermore, the accumulation of mucus and the influence of vibration frequency on its rheological properties (10) are obvious and they could have an effect on the shear waves. Upcoming studies will be devoted to conduct another numerical study to illustrate the deformation of the airways subjected to vibrations by a simplified two-phase flow model, including both mucus and air.

## Data Availability

The datasets presented in this study can be found in online repositories. The names of the repository/repositories and accession number(s) can be found in the article/Supplementary Material.

## Glossary

A: specific area of the porous section (m²)
c: wave speed (m.s^−1^)
d: diameter (m)
E: Young's Modulus (Pa)
f: frequency (Hz)
F: force per unit volume (N.m^−3^)
*F*(*Θ*): frequency correction function
J: Bessel function
K: bulk modulus (Pa.s)
k: wave number
u: steady-state dynamic oscillatory displacement (m)
p: dynamic pressure (Pa)
R: lung parenchyma parameters with respect to the air inside
r: rate of introduction of the gas (m^−3^)
*T*(*Θ*): frequency correction factor
t: time (s)
x: displacement in the x direction (m)

## Greek letters

*α, β*: lung parenchyma parameters with respect to the air inside
*Γ*, Δ, *ε*, *Ω*: determined constants for the quadruple equation
*κ*: permeability (m^2^)
*λ*: Lamé's parameter (Pa)
*µ*: shear modulus (Pa.s)
*ν*: Poisson's ratio
*η*: dynamic viscosity (Pa.s)
ξ: sinuosity function
*ρ*: density (kg.m^−3^)
*τ*: tortuosity
φ: air volume fraction
*ω*: angular frequency (s^−1^)
Θ: frequency parameter

## Subscript

e: external
f: fluid
g: gas
l: lungs
o: osseous region
p: porous medium
pf: fast compression
ps: slow compression
s: shear
sk: skeleton
st: soft tissue
*i*: Spatial coordinate index (e.g., *x*, *y*, *z*)
*j*: Spatial coordinate index for first derivative
*jj*: Second-order spatial derivative (Laplacian or strain component)
*ii*: Second-order spatial derivative (Laplacian or divergence)
,*i*: First-order derivative with respect to *x_i_*
,*ii*: Second-order derivative with respect to *x_i_* (Laplacian of scalar fields like pressure)

## References

[1] Tikka T Hilmi OJ. Upper airway tract complications of endotracheal intubation. Br J Hosp Med. (2019) 80:441–7. 10.12968/hmed.2019.80.8.441 31437047

[2] Russotto V Myatra SN Laffey JG Tassistro E Antolini L Bauer P Intubation practices and adverse peri-intubation events in critically ill patients from 29 countries. J Am Med Assoc. (2021) 325:1164–72. 10.1001/jama.2021.1727 PMC7988368 33755076

[3] Sundaresan A Chase G Hann J and Shaw CE M G. Dynamic functional residual capacity can be estimated using a stress–strain approach. Comput Methods Programs Biomed. (2011) 101:135–43. 10.1016/j.cmpb.2010.05.005 20538364

[4] Matettore A Ramnarayan P Jones A Randle E Lutman D O’Connor M Adverse tracheal intubation-associated events in pediatric patients at nonspecialist centers: a multicenter prospective observational study. Pediatr Crit Care Med. (2019) 20:518–26. 10.1097/PCC.0000000000001923 30946293

[5] Mauri T Yoshida T Bellani G Goligher EC Carteaux G Rittayamai N Esophageal and transpulmonary pressure in the clinical setting: meaning, usefulness and perspectives. Intensive Care Med. (2016) 42:1360–73. 10.1007/s00134-016-4400-x 27334266

[6] Cakmak A Ince I Saglam D Savci M V S Yagli N Physiotherapy and rehabilitation implementation in intensive care units: a survey study. Turk Thorac J. (2019) 20:114–9. 10.5152/TurkThoracJ.2018.18107 30958983 PMC6453635

[7] Uzundurukan A Poncet S Boffito DC Micheau P. Acoustic airway clearance devices: a systematic review of experimental and numerical studies. Biomed Eng Adv. (2024a) 8:100134. 10.1016/j.bea.2024.100134

[8] Spapen HD De Regt J Honoré PM. Chest physiotherapy in mechanically ventilated patients without pneumonia—a narrative review. J Thorac Dis. (2017) 9:E44–9. 10.21037/jtd.2017.01.32 28203436 PMC5303101

[9] Mefford GW. Biphasic cuirass ventilation for airway secretions. In: Esquinas AM, editor. Humidification in the Intensive Care Unit: The Essentials. Cham: Springer International Publishing (2023). p. 313–21. 10.1007/978-3-031-23953-3_34

[10] Schieppati D Germon R Galli F Rigamonti MG Stucchi M Boffito DC. Influence of frequency and amplitude on the mucus viscoelasticity of the novel mechano-acoustic Frequencer^TM^. Respir Med. (2019) 153:52–9. 10.1016/j.rmed.2019.04.011 31163350

[11] Uzundurukan A Poncet S Boffito DC Micheau P. CT-FEM of the human thorax: frequency response function and 3D harmonic analysis at resonance. Comput Methods Programs Biomed. (2024b) 246:108062. 10.1016/j.cmpb.2024.108062 38359553

[12] Bruner MM Bazan C Liu B Cheng C Chad M Sievert C Effects of high frequency chest wall oscillation (HFCWO) on clinical symptoms in COPD. Res. Sq. (2024):rs.3.rs–4165729. 10.21203/rs.3.rs-4165729/v1 38659871 PMC11042428

[13] Hansen LG Warwick WJ. High-frequency chest compression system to aid in clearance of mucus from the lung. Biomed Instrum Technol. (1990) 24:289–94. 2390665

[14] Hsia CCW Hyde DM Weibel ER. Lung structure and the intrinsic challenges of gas exchange. Compr Physiol. (2016) 6:827–95. 10.1002/cphy.c150028 27065169 PMC5026132

[15] Vasquez PA Forest MG. Complex fluids and soft structures in the human body. In: Spagnolie SE, editor. Complex Fluids in Biological Systems: Experiment, Theory, and Computation. New York, NY: Springer (2015). p. 53–110. 10.1007/978-1-4939-2065-5_2

[16] Faffe D. S., and Zin W. A. (2009). Lung parenchymal mechanics in health and disease. Physiol Rev*.* 89, 759–75. 10.1152/physrev.00019.2007 19584312 PMC7203567

[17] Ebihara T Venkatesan N Tanaka R Ludwig MS. Changes in extracellular matrix and tissue viscoelasticity in bleomycin-induced lung fibrosis. Temporal aspects. Am J Respir Crit Care Med. (2000) 162:1569–76. 10.1164/ajrccm.162.4.9912011 11029378

[18] Salerno FG Ludwig MS. Elastic moduli of excised constricted rat lungs. J Appl Physiol. (1999) 86:66–70. 10.1152/jappl.1999.86.1.66 9887114

[19] Ito S Ingenito EP Brewer KK Black LD Parameswaran H Lutchen KR Mechanics, nonlinearity, and failure strength of lung tissue in a mouse model of emphysema: possible role of collagen remodeling. J Appl Physiol. (2005) 98:503–11. 10.1152/japplphysiol.00590.2004 15465889

[20] Kumar AK Jain S Jain S Ritam M Xia Y Chandra R. Physics-informed neural entangled-ladder network for inhalation impedance of the respiratory system. Comput Methods Programs Biomed. (2023) 231:107421. 10.1016/j.cmpb.2023.107421 36805280

[21] Barahona J Sahli Costabal F Hurtado DE. Machine learning modeling of lung mechanics: assessing the variability and propagation of uncertainty in respiratory-system compliance and airway resistance. Comput Methods Programs Biomed. (2024) 243:107888. 10.1016/j.cmpb.2023.107888 37948910

[22] Knopp JL Chase JG Kim KT Shaw GM. Model-based estimation of negative inspiratory driving pressure in patients receiving invasive NAVA mechanical ventilation. Comput Methods Programs Biomed. (2021) 208:106300. 10.1016/j.cmpb.2021.106300 34348200

[23] Williams EC Motta-Ribeiro GC Vidal Melo MF. Driving pressure and transpulmonary pressure: how do we guide safe mechanical ventilation? Anesthesiology. (2019) 131:155–63. 10.1097/ALN.0000000000002731 31094753 PMC6639048

[24] Bersten AD. 33—acute Respiratory distress syndrome. In: Bersten AD Soni N, editors. Oh’s Intensive Care Manual. Boston: Butterworth-Heinemann (2014). p. 382–391.e4. 10.1016/B978-0-7020-4762-6.00033-3

[25] Mead J. Mechanical poperties of lungs. Physiol. Rev. (1961) 41:281–330. 10.1152/physrev.1961.41.2.281 13768766

[26] Jadeh M Lai-Fook SJ Bhagat PK Kraman SS. Propagation of stress waves in inflated sheep lungs. J Appl Physiol. (1989) 66(6):2675–80. 10.1152/jappl.1989.66.6.2675 2745329

[27] Dai Z Peng Y Mansy HA Sandler RH Royston TJ. Comparison of poroviscoelastic models for sound and vibration in the lungs. J Vib Acoust. (2014b) 136:050905. 10.1115/1.4026436 PMC4112928 25278740

[28] Guarino JR. Auscultatory percussion of the chest. Lancet. (1980) 1:1332–4. 10.1016/s0140-6736(80)91788-2 6104133

[29] Dai Z Peng Y Henry BM Mansy HA Sandler RH Royston TJ. A comprehensive computational model of sound transmission through the porcine lung. J Acoust Soc Am. (2014a) 136:1419–29. 10.1121/1.4890647 25190415 PMC4165230

[30] Royston T Zhang X Mansy H Sandler R. Modeling sound transmission through the pulmonary system and chest with application to diagnosis of a collapsed lung. J Acoust Soc Am. (2002) 111:1931–46. 10.1121/1.1452742 12002875

[31] Rice DA. Sound speed in pulmonary parenchyma. J Appl Physiol. (1983) 54:304–8. 10.1152/jappl.1983.54.1.304 6826415

[32] Wodicka GR Stevens KN Golub HL Cravalho EG Shasnnon DC. A model of acoustic transmission in the respiratory system. IEEE Trans Biomed Eng. (1989) 36:925–34. 10.1109/10.35301 2777281

[33] Biot MA. Theory of propagation of elastic waves in a fluid-saturated porous solid. I. Low-frequency range. J Acoust Soc Am. (1956a) 28:168–78. 10.1121/1.1908239

[34] Biot MA. Theory of propagation of elastic waves in a fluid-saturated porous solid. II. Higher Frequency Range. J Acoust Soc Am. (1956b) 28:179–91. 10.1121/1.1908241

[35] Goodwin M. Measurement of resonant frequencies in the human chest. Proc Inst Mech Eng H J Eng Med. (1994) 208:83–9. 10.1243/PIME_PROC_1994_208_269_02

[36] Ong J Ghista D. Applied chest-wall vibration therapy for patients with obstructive lung disease. Hum Resp An Phy Math Mod Num Sim App. (2006) 3:157–67. 10.2495/978-1-85312-944-5/08

[37] Parreira VF Delguste P Jounieaux V Aubert G Dury M Rodenstein DO. Glottic aperture and effective minute ventilation during nasal two-level positive pressure ventilation in spontaneous mode. Am J Respir Crit Care Med. (1996) 154:1857–63. 10.1164/ajrccm.154.6.8970381 8970381

[38] Naue WDS Da Silva ACT Güntzel AM Condessa RL De Oliveira RP Rios Vieira SR. Increasing pressure support does not enhance secretion clearance if applied during manual chest wall vibration in intubated patients: a randomised trial. J Physiother. (2011) 57:21–6. 10.1016/S1836-9553(11)70003-0 21402326

[39] Siklosi M Jensen OE Tew RH Logg A. Multiscale modeling of the acoustic properties of lung parenchyma. ESAIM Proc. (2008) 23:78–97. 10.1051/proc:082306

[40] Peng Y Dai Z Mansy HA Royston TJ. Poro-Visco-Elastic modeling of mechanical wave motion in the lungs. ASME 2012 Summer Bioengineering Conference, Parts A and B; Fajardo, Puerto Rico, USA: American Society of Mechanical Engineers (2012). p. 751–2. 10.1115/SBC2012-80544

[41] Schanz M. In: Schanz M, editors. Wave Propagation. Berlin, Heidelberg: Springer (2001) p. 105–34. 10.1007/978-3-540-44575-3_7

[42] Fields DA Higgins PB Hunter GR. Assessment of body composition by air-displacement plethysmography: influence of body temperature and moisture. Dyn Med. (2004) 3:3. 10.1186/1476-5918-3-3 15059287 PMC411054

[43] Bonnet G. Basic singular solutions for a poroelastic medium in the dynamic range. J Acoust Soc Am. (1987) 82:1758–62. 10.1121/1.395169

[44] Roth S Torres F Feuerstein P Thoral-Pierre K. Anthropometric dependence of the response of a thorax FE model under high speed loading: validation and real world accident replication. Comput Methods Programs Biomed. (2013) 110:160–70. 10.1016/j.cmpb.2012.11.004 23246086

[45] González-Suárez A Irastorza RM Deane S O’Brien B O’Halloran M Elahi A. Full torso and limited-domain computer models for epicardial pulsed electric field ablation. Comput Methods Programs Biomed. (2022) 221:106886. 10.1016/j.cmpb.2022.106886 35597202

[46] Uzundurukan A Poncet S Boffito DC Micheau P. Realistic 3D CT-FEM for target-based multiple organ inclusive studies. J Biomed Eng Biosci. (2023) 10:24–35. 10.11159/jbeb.2023.005

[47] Peng Y Dai Z Mansy HA Sandler RH Balk RA Royston TJ. Sound transmission in the chest under surface excitation: an experimental and computational study with diagnostic applications. Med Biol Eng Comput. (2014) 52:695–706. 10.1007/s11517-014-1172-8 25001497 PMC4160106

[48] Nuyts J Dupont P Stroobants S Benninck R Mortelmans L Suetens P. Simultaneous maximum a posteriori reconstruction of attenuation and activity distributions from emission sinograms. IEEE Trans Med Imaging. (1999) 18:393–403. 10.1109/42.774167 10416801

[49] Wilson MS Wynn TA. Pulmonary fibrosis: pathogenesis, etiology and regulation. Mucosal Immunol. (2009) 2:103–21. 10.1038/mi.2008.85 19129758 PMC2675823

[50] Ganesan S Man CS Lai-Fook SJ. Generation and detection of lung stress waves from the chest surface. Respir Physiol. (1997) 110:19–32. 10.1016/s0034-5687(97)00065-0 9361149

[51] Hill DB Vasquez PA Mellnik J McKinley SA Vose A Mu F A biophysical basis for mucus solids concentration as a candidate biomarker for airways disease. PLoS One. (2014) 9:e87681. 10.1371/journal.pone.0087681 24558372 PMC3928107

[52] Von Gierke HE Oestreicher HL Franke EK Parrack HO Von Wittern WW. Physics of vibrations in living tissues. J Appl Physiol. (1952) 4:886–900. 10.1152/jappl.1952.4.12.886 14946086

[53] Bonnet G Auriault J-L. Dynamics of saturated and deformable porous media. In: Boccara N Daoud M, editors. Physics of Finely Divided Matter. Berlin, Heidelberg: Springer Berlin Heidelberg (1985). p. 306–16. 10.1007/978-3-642-93301-1_37

[54] Hajari AJ Yablonskiy DA Sukstanskii AL Quirk JD Conradi MS Woods JC. Morphometric changes in the human pulmonary acinus during inflation. J Appl Physiol. (2012) 112:937–43. 10.1152/japplphysiol.00768.2011 22096115 PMC3311655

