# Supplementary material for: Effect of the transpulmonary pressure on the lungs’ vibroacoustic response: a first numerical perspective
Source: Front Digit Health. 2025 Apr 4;7:1434578. doi: 10.3389/fdgth.2025.1434578 (PMC12006108; doi:10.3389/fdgth.2025.1434578)
Supplement: Supplementary file 1 [file Table1.pdf]

Effect of the transpulmonary pressure on the lungs' vibroacoustic response: A first numerical perspective

Arife Uzundurukan[*1,2], Sébastien Poncet[1], Daria Camilla Boffito[2], Philippe Micheau[1]

[1]Centre de Recherche Acoustique-Signal-Humain, Université de Sherbrooke, 2500 Bd de l'Université, Sherbrooke, J1K 2R1, QC, CA
[2]Department of Chemical Engineering, École Polytechnique de Montréal, 2500 Chem. de Polytechnique, Montréal, H3T 1J4, QC, CA
[*]corresponding author: Arife.Uzundurukan@Usherbrooke.ca

Supplementary Material

Supplementary Table 1. Analytically calculated compression (slow and fast) and shear wave speeds according to the frequency and transpulmonary pressures.

Transpulmonary Pressure (cm H_2O)	Frequency (Hz)	Compression Wave Speeds (m/s)		Shear Wave Speed (m/s)
		Slow	Fast	
20	5	1.956164914227140 + 0.710013256174307i	23.559779988151190 + 1.075342196975312i	2.106079780170350 + 0.016953938524727i
	10	2.293688895308498 + 0.790788688728530i	24.021017172110714 + 1.496740112613916i	2.113157743903338 + 0.023896211120012i
	15	2.510274398124842 + 0.833038864012230i	24.3740085058756 + 1.81092091212329i	2.118587814404182 + 0.029191777533957i
	20	2.671845763041789 + 0.859303643161530i	24.6708080599961 + 2.06976393744426i	2.12316471462635 + 0.0336351247173521i
	25	2.801230538381723 + 0.876943624213951i	24.931602179812190 + 2.293349747721800i	2.127196296165582 + 0.037533967884053i
	30	2.909275278219610 + 0.889281812491933i	25.166756203265130 + 2.491969255112704i	2.130840455004777 + 0.041046111008216i
	35	3.00205136556606 + 0.898091869110611i	25.3824353407555 + 2.67173093652475i	2.134190994974571 + 0.044265290383371i
	40	3.08332435445418 + 0.904425023371188i	25.582661739956656 + 2.836611470579920i	2.137309038567501 + 0.047252576181348i
	45	3.15560112821208 + 0.908951031192551i	25.7702328564652 + 2.98937060353671i	2.14023704444408 + 0.0500503849749211i
	50	3.22063980238507 + 0.912119862231210i	25.9471882002417 + 3.13201578715253i	2.143005928547375 + 0.052689597894830i
	55	3.27972362970436 + 0.914246374142937i	26.1150694880093 + 3.26606096932040i	2.145639031458444 + 0.055193526102758i
	60	3.333819397586114 + 0.915558087370857i	26.275076077025354 + 3.392681099899113i	2.148154487679881 + 0.057580278811472i
	65	3.38367446410820 + 0.916223756730390i	26.4281630202876 + 3.51280952639109i	2.15056671957502 + 0.0598642563400497i

	70	3.42987902571544 + 0.916371277890519i	26.5751056929449 + 3.62720213540995i	2.15288742150474 + 0.0620571336110593i
	75	3.47290762937916 + 0.916099350676681i	26.7165439505722 + 3.73648114427498i	2.15512623192708 + 0.0641685317742730i
	80	3.51314774769793 + 0.915485325177795i	26.8530132285100 + 3.84116591653535i	2.15729120648558 + 0.0662064909326018i
	85	3.55091999173243 + 0.914590626881996i	26.9849670155329 + 3.94169521155428i	2.15938915969612 + 0.0681778115474747i
	90	3.58649274816435 + 0.913464597584853i	27.1127934587680 + 4.03844360946742i	2.16142591726530 + 0.0700883065346966i
	95	3.62009299762695 + 0.912147271211854i	27.2368278719815 + 4.13173387279126i	2.16340650605047 + 0.0719429910460312i
	100	3.65191445542638 + 0.910671416475439i	27.3573623194821 + 4.22184640922196i	2.16533529952335 + 0.0737462277878251i
10	5	1.14744570384360 + 0.424709366959034i	21.5679782902268 + 0.830298571739725i	1.358538850347924 + 0.004165856001022i
	10	1.34859877205312 + 0.477624432531827i	21.9232417227673 + 1.15667860256306i	1.36026965651420 + 0.00588391382947527i
	15	1.47869881521936 + 0.506755978443237i	22.1950403892045 + 1.40054254530088i	1.361597713239849 + 0.007199264509671i
	20	1.57639024607609 + 0.525790921062567i	22.4235299795371 + 1.60184406741072i	1.36271728556145 + 0.00830616486971696i
	25	1.65507146725782 + 0.539261071394086i	22.6242835150940 + 1.77603907626259i	1.36370362118697 + 0.00927985787355789i
	30	1.72111641370683 + 0.549238495264107i	22.8052970486801 + 1.93104441094365i	1.36459531210565 + 0.0101589323095752i
	35	1.77809720469792 + 0.556842856225990i	22.9713259423795 + 2.07155716882210i	1.36541528395312 + 0.0109663114635745i
	40	1.82823203035018 + 0.562745894996974i	23.1254719774415 + 2.20063392271010i	1.36617847419378 + 0.0117169309793184i
	45	1.87299947875265 + 0.567380829252470i	23.2698919707813 + 2.32039508190412i	1.36689525852569 + 0.0124211650901054i
	50	1.91343783254662 + 0.571042297209515i	23.4061579370778 + 2.43238252513254i	1.36757319150389 + 0.0130865671392455i
	55	1.95030592553880 + 0.573939031124756i	23.5354578403032 + 2.53775873983588i	1.36821797626178 + 0.0137188392359524i
	60	1.98417621337140 + 0.576223764901007i	23.6587155385087 + 2.63742568318829i	1.36883404360900 + 0.0143224113093104i
	65	2.01549181954347 + 0.578011233386358i	23.7766664502216 + 2.73209968897208i	1.36942491716946 + 0.0149008062155438i
	70	2.04460316121671 + 0.579389524481335i	23.8899074234607 + 2.82236078608315i	1.36999345391107 + 0.0154568802455272i

	75	2.07179237991098 + 0.580427520403463i	23.9989308089911 + 2.90868636153028i	1.37054200840745 + 0.0159929873698986i
	80	2.09729016783250 + 0.581179935684131i	24.1041484556345 + 2.99147484279711i	1.37107254845815 + 0.0165110948467539i
	85	2.12128767686751 + 0.581690823205810i	24.2059090489525 + 3.07106279301299i	1.37158673859317 + 0.0170128667162769i
	90	2.14394514749741 + 0.581996072644122i	24.3045109209700 + 3.14773752791728i	1.37208600173511 + 0.0174997254552372i
	95	2.16539829020798 + 0.582125228018402i	24.4002116985840 + 3.22174660939117i	1.37257156562101 + 0.0179728983925901i
	100	2.18576309035780 + 0.582102834116706i	24.4932356953590 + 3.29330511116787i	1.37304449834942 + 0.0184334532514922i
